# Probiotic properties of *E* *nterococcus* strains isolated from traditional naturally fermented cream in China

**DOI:** 10.1111/1751-7915.12306

**Published:** 2015-07-22

**Authors:** Lidong Guo, Tingting Li, Yaru Tang, Lijie Yang, Guicheng Huo

**Affiliations:** ^1^ Key Laboratory of Dairy ScienceMinistry of EducationNortheast Agricultural UniversityHarbinChina; ^2^ College of PharmacyHeilongjiang University of Chinese MedicineHarbinChina

## Abstract

The purpose of this study was to evaluate the probiotic properties of *Enterococcus* strains isolated from traditional naturally fermented cream in China. Four *E* *nterococcus* isolates showed high cholesterol removal ability in media were identified as *E* *nterococcus durans* (KLDS 6.0930 and 6.0933) and *E* *nterococcus faecalis* (KLDS 6.0934 and 6.0935) by 16S rRNA and pheS gene sequences, respectively, and selected for further evaluation. In order to assess the probiotic potential and safety of these strains, the property of four *E* *nterococcus* strains were examined, including acid and bile tolerance, adherence to Caco‐2 cells and antibiotics susceptibility. All four strains showed potential cholesterol assimilation, de‐conjugation of bile salts and/or cholesterol degradation to remove cholesterol *in vitro*. In addition, the potential effect of *E*. *durans* KLDS 6.0930 on serum cholesterol levels was evaluated in Sprague‐Dawley rats. After 4 weeks administration, compared with rats fed a high‐cholesterol diet without lactic acid bacteria supplementation, there was a significant (*P* < 0.05) decrease in the total cholesterol and low‐density lipoprotein cholesterol levels in the serum of rats treated with KLDS 6.0930. Furthermore, total bile acid level in the feces was significantly (*P* < 0.05) increased after KLDS 6.0930 administration. These observations suggested that the strain *E*. *durans* KLDS 6.0930 may be used in the future as a good candidate for lowering human serum cholesterol levels.

## Introduction

Cardiovascular disease (CVD) is a leading cause of death worldwide, and over 80% of CVD deaths take place in low and middle‐income countries (WHO, 2011). Elevated serum cholesterol level is widely recognized as a contributory risk factor for the development of CVD, such as coronary heart disease (CHD), etc. Numerous cholesterol‐lowering drugs have been developed, the best example being the statins which are 3‐hydroxy‐3methylglutaryl coenzyme A reductase inhibitors. However, all the statins have severe side effects, particularly in a long‐term therapeutic use (Elis and Lishner, 2012; Gotto and Moon, 2012). Thus, a number of non‐pharmacological approaches (including dietary ones) meant to lower cholesterol levels were designed and investigated (Deng, 2009; Goto *et al*., 2013; Kim *et al*., 2013). Various dietary approaches have been used to alleviate this case, including the use of probiotics, which provided health benefits to the host when administered in adequate amounts (FAO/WHO, 2001). The main probiotics are lactic acid bacteria, such as *Lactobacillus*, *Bifidobacterium* and *Enterococcus*, which are inherent members in the gastrointestinal tract of human and animals. In general, these probiotic bacteria are also predominant organisms in the traditional fermented dairy products, such as *Jiaoke*, which is a homemade naturally fermented cream in Inner Mongolia of China (Guo *et al*., 2011). Various strains of *Lactobacillus* and *Bifidobacterium* have been suggested to lower cholesterol levels *in vitro* or *in vivo* by different mechanisms (Huang *et al*., 2010; 2014; Jeun *et al*., 2010; Ooi and Liong, 2010; Ahire *et al*., 2012; Bordoni *et al*., 2013; Ebel *et al*., 2014; Gorenjak *et al*., 2014; Kim *et al*., 2014). In contrast, more limited and sporadic data concerning cholesterol‐lowering activity by *Enterococcus* strains are available (Agerbaek *et al*., 1995; Agerholm‐Larsen *et al*., 2000; Rossi *et al*., 2000; Hlivak *et al*., 2005), particularly in cholesterol‐lowering mechanisms.

The aims of the present study were to select probiotic *Enterococcus* strains from the *Jiaoke* based on the cholesterol‐reducing activity, and to determine the cholesterol removal mechanisms of these strains from media, and then to evaluate their acid and bile tolerance, adhesion abilities to Caco‐2 cells and antibiotics susceptibility. A strain of *Enterococcus* showing desirable properties was further screened for assay of its cholesterol‐lowering effects in rats.

## Results

### Screening of cholesterol‐lowering isolates

In total, 23 strains of Gram‐positive, catalase‐negative, globular‐shaped isolates were obtained from 20 samples of *Jiaoke*. Four isolates were selected for further research based on the cholesterol removal ratio. Table 1 shows the cholesterol removal ability of the four isolates in media. The cholesterol removal of KLDS 6.0930 from media was higher than other isolates. In the presence of KLDS 6.0930, 6.0933, 6.0934 and 6.0935, 56.61%, 46.99%, 41.29% and 52.01% of cholesterol disappeared from supernatant fluids respectively. A part of cholesterol was precipitated and re‐solubilized in the washing fluid (13.33%, 9.38%, 23.57% and 9.40%, respectively), and an amount of cholesterol (14.50%, 10.67%, 17.72% and 16.88%, respectively) was retained in the bacterial cells, and yet another part was not recovered except for KLDS 6.0934.

**Table 1:** Cholesterol removal ability of *E* *nterococcus* strains

Strains	Cholesterol (%)*			CDR (%)**	CR (%)***
	Supernatant fluid	Washing fluid	Fragmentized‐cells solution		
KLDS 6.0930	43.39 ± 2.00^d^	13.33 ± 1.54^b^	14.50 ± 1.28^b^	28.78 ± 2.05^a^	56.61 ± 2.00^a^
KLDS 6.0933	53.01 ± 1.67^b^	9.38 ± 1.03^c^	10.67 ± 1.07^c^	26.93 ± 2.73^a^	46.99 ± 1.67^c^
KLDS 6.0934	58.71 ± 1.72^a^	23.57 ± 1.02^a^	17.72 ± 1.18^a^	0.01 ± 3.89^b^	41.29 ± 1.72^d^
KLDS 6.0935	47.99 ± 1.59^c^	9.40 ± 1.42^c^	16.88 ± 1.23^a^	25.72 ± 2.73^a^	52.01 ± 1.59^b^

* The amount of cholesterol in the different fractions is expressed as a percentage of the initial concentration of cholesterol in the medium.

** The amount of cholesterol not recovered is expressed as a percentage of the initial concentration of cholesterol in the medium.

*** The amount of cholesterol lost from supernatant fluid is expressed as a percentage of the initial concentration of cholesterol in the medium.

Values are expressed as mean ± standard deviation.

^a^^,^^b^^,^^c^^,^^d^ Means in the same column followed by different lowercase letters are significantly different (*P* < 0.05).

### Identification of the strains

The phylogenetic trees based on the 16S rRNA and phenylalanyl‐tRNA synthase alpha chain (*pheS*) gene sequences were created (see Fig. S1 and Fig. S2 in ‘Supplementary materials’). These results showed that the KLDS 6.0930 and KLDS 6.0933 isolates were identified by 16S rRNA and *pheS* gene sequences as *Enterococcus durans*, while the KLDS 6.0934 and KLDS 6.0935 isolates were *Enterococcus faecalis*.

### Acid tolerance

**Figure 1:**
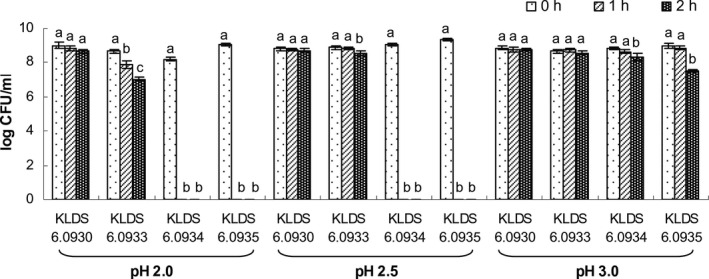
Effect of pH 2.0, pH 2.5 and pH 3.0 on viability of *E* *nterococcus* strains. The results are expressed as mean ± standard deviation.

The effect of an acidic pH (2.0, 2.5 and 3.0) on the survival of the *Enterococcus* strains over different incubation periods was shown in Fig. 1. All four strains were strongly tolerant to pH 3.0, and could survive for 2 h. However, no growth were observed in the *E. faecalis* KLDS 6.0934 and KLDS 6.0935 strains at pH 2.0 or pH 2.5 after 1 h, which showed that the two strains were sensitive to these acid conditions. In contrast, the viable counts of *E. durans* KLDS 6.0930 and KLDS 6.0933 strains remained 10^7^ cfu ml^‐1^ after 2 h of incubation at pH 2.0 or pH 2.5. Compared with all other strains, the *E. durans* KLDS 6.0930 was the most acid‐tolerant strain, whose viability at pH 2.0 was not significantly decreased (*P* > 0.05) after 2 h.

### Bile tolerance

The effect of bile on the growth of isolates was evaluated and shown in Table 2. The growth of *Enterococcus* strains in de Man, Ragosa and Sharpe (MRS) broth without bile was used as control. The data exhibited that *E. durans* KLDS 6.0930 strain was better than other isolates growth in both MRS broth and MRS broth supplemented with bile. The times required to increase the A620 nm reading by 0.3 units were 3.9 h and 4.7 h when KLDS 6.0930 was grown in MRS broth and MRS broth plus 0.3% oxgall respectively. The significant differences (*P* < 0.05) were observed among the four strains in the tolerance to bile.

**Table 2:** Effect of bile on the growth rate of *E* *nterococcus* strains

Strains	T (h)*	
	MRS broth	MRS broth + 0.3 % oxgall
KLDS 6.0930	3.9 ± 0.1^[Link]^, ^[Link]^	4.7 ± 0.1^[Link]^, ^[Link]^
KLDS 6.0933	5.7 ± 0.2^[Link]^, ^[Link]^	5.4 ± 0.2^[Link]^, ^[Link]^
KLDS 6.0934	5.3 ± 0.2^[Link]^, ^[Link]^	5.8 ± 0.3^[Link]^, ^[Link]^
KLDS 6.0935	5.0 ± 0.2^[Link]^, ^[Link]^	5.9 ± 0.3^[Link]^, ^[Link]^

* Time (h) required for absorbance at 620 nm to increase by 0.3 units in each medium.

Values are expressed as means ± standard deviation.

^a^^,^^b^^,^^c^ Means in the same column followed by different lowercase letters are significantly different (*P* < 0.05).

^A^^,^^B^ Means in the same row followed by different uppercase letters are significantly different (*P* < 0.05).

### Adherence to Caco‐2 cells in vitro

**Figure 2:**
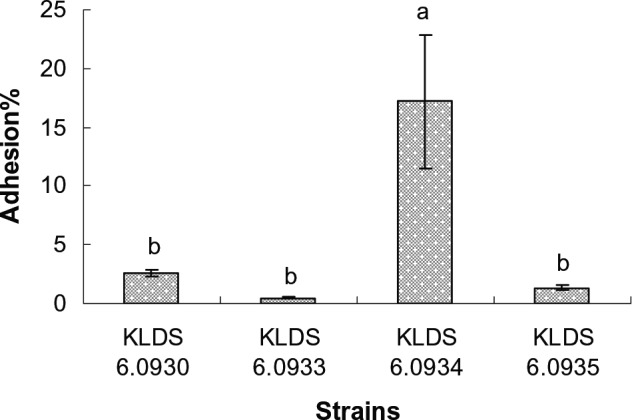
Adhesion ability of *E* *nterococcus* isolates to Caco‐2 epithelial cells. The results are expressed as means ± standard deviation, and the different letters (a, b) represent significant differences (*P* < 0.05).

All the *Enterococcus* strains were examined for their ability to adhere to Caco‐2 cells. The results were shown in Fig. 2. In general, the bacteria showed strain dependent in adhesion to Caco‐2 cells. The strain KLDS 6.0934 exhibited significantly (*P* < 0.05) stronger adhesion ability than other isolates, and the adhesion rate was 17.2%. The adhesion rate of strains KLDS 6.0930, KLDS 6.0933 and KLDS 6.0935 were 1.6%, 0.5% and 1.3% respectively.

### Antibiotic susceptibility tests

The resistance levels to antibiotic were detected for the four isolates and were shown in Table 3. According to the breakpoints set by European Food Safety Authority (2008), all tested strains were found to be susceptible to ampicillin, erythromycin and penicillin G, but were resistant to gentamycin, with minimum inhibitory concentration (MIC) values of over 1024 μg ml^‐1^. Regarding the vancomycin, *E. durans* KLDS 6.0930 and KLDS 6.0933 were found to be susceptible, while *E. faecalis* KLDS 6.0934 and KLDS 6.0935 were not completely inhibited at 1024 μg ml^‐1^. Moreover, the two *E. faecalis* strains could be inhibited by chloramphenicol, while the two *E. durans* strains could be completely inhibited at 16 μg ml^‐1^. Subsequently, all antibiotic‐resistant strains were investigated for the presence of antibiotic‐resistance genes by polymerase chain reaction (PCR)‐based detection technology. From the results of PCR amplification, none of the tested antibiotic‐resistance gene was detected in all experimental strains.

**Table 3:** Antibiotics susceptibility of *E* *nterococcus* strains

Stains	MICs (μg ml^‐1^)					
	ampicillin	vancomycin	gentamycin	erythromycin	chloramphenicol	penicillin
*E. durans* KLDS 6.0930	<1	2	>1024^a^	2	16^a^	<1
*E. durans* KLDS 6.0933	<1	4	>1024^a^	<1	16^a^	<1
*E. faecalis* KLDS 6.0934	<1	>1024^a^	>1024^a^	<1	8	<1
*E. faecalis* KLDS 6.0935	<1	>1024^a^	>1024^a^	<1	8	<1

a Resistant according to the European Food Safety's breakpoints (European Food Safety Authority, 2008).

MICs, minimum inhibitory concentrations; R, Resistant.

### Body weight and food efficiency

All the experimental rats were generally healthy throughout the feeding trial period, and their body weight gain, food intake and food efficiency were recorded and calculated for all the groups after 28 day gavage as indicated in Table 4. Statistical analysis suggested that no significant differences (*P* > 0.05) was observed on body weight gain, food intake or food efficiency. This indicates that all rats grew in similar patterns.

**Table 4:** The body weight, food intake and food efficiency of rats fed different diets

	ND‐C	HD‐C	HD‐KLDS 6.0930
Body weight gain (g)	203.5 ± 15.7^a^	194.6 ± 14.5^a^	199.0 ± 15.6^a^
Total food intake (g)	523.6 ± 12.3^a^	513.2 ± 17.2^a^	518.0 ± 8.1^a^
Food efficiency (%)*	38.9 ± 3.3^a^	37.9 ± 2.8^a^	38.4 ± 3.2^a^

Values are expressed as mean ± standard deviation, *n* = 8.

* Food efficiency (%) = (body weight gain/food intake) × 100.

a Means within a row differ insignificantly (*P* > 0.05).

ND‐C, normal diet control; HD‐C, high‐cholesterol diet control; HD‐KLDS 6.0930, HD supplemented with *Enterococcus durans* KLDS 6.0930.

### Serum lipid profile

**Figure 3:**
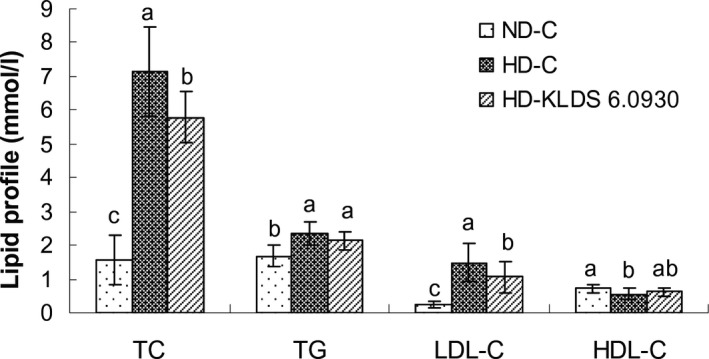
Total cholesterol, TG, LDL‐C and HDL‐C levels in the serum of rats fed a different diet for 4 weeks. ND‐C, normal diet control; HD‐C, high‐cholesterol diet control; HD‐KLDS 6.0930, HD supplemented with *E* *nterococcus durans* KLDS 6.0930. The results are expressed as means ± standard deviation, *n* = 8. Means within the same lipid series with different lowercase letters (a, b, c) are significantly different (*P* < 0.05).

The effect of dietary treatments on serum lipid profile, including total cholesterol (TC), triglyceride (TG), low‐density lipoprotein cholesterol (LDL‐C) and high‐density lipoprotein cholesterol (HDL‐C), was shown in Fig. 3. Rats fed the KLDS 6.0930 diet had significantly (*P* < 0.05) lower serum TC and LDL‐C levels compared with the high‐cholesterol diet control group. The reduction in TC and LDL‐C levels was 19.1% and 27.7% in the KLDS 6.0930 group respectively. However, no significant differences (*P* > 0.05) were observed in serum TG and HDL‐C levels between the KLDS 6.0930 group and high‐cholesterol diet control group.

### Fecal total bile acid excretions

**Figure 4:**
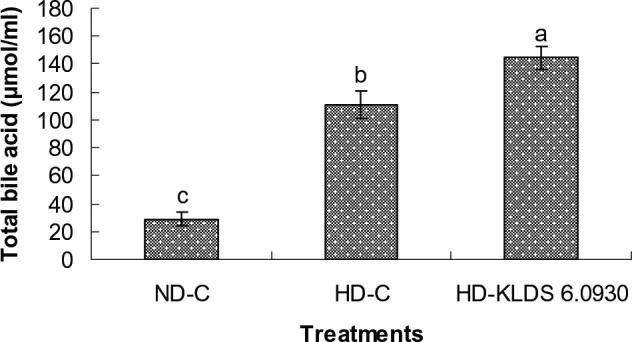
Fecal bile acid concentrations in rats fed a different diet for 4 weeks. ND‐C, normal diet control; HD‐C, high‐cholesterol diet control; HD‐KLDS 6.0930, HD supplemented with *E* *nterococcus durans* KLDS 6.0930. The results are expressed as the means ± standard deviation. Means with different letters (a, b, c) differ significantly (*P* < 0.05).

As shown in Fig. 4, significant difference of fecal total bile acid level was observed among the various groups (*P* < 0.05). The fecal total bile acid excretion in rats fed the KLDS 6.0930 diet was significantly higher than that of other groups. Moreover, the fecal bile acid content in rats fed on the normal diet was significantly lower than that of the high‐cholesterol diet control group (*P* < 0.05).

## Discussion

Accumulating evidence suggests that various probiotic bacteria, such as *Lactobacillus* and *Bifidobacterium*, have exerted cholesterol‐lowering activity *in vitro* or *in vivo*, but few of *Enterococcus* strains was reported. For this reason, four *Enterococcus* strains isolated from *Jiaoke* were screened on the basis of the cholesterol removal ability in media, and identified as *E. durans* (KLDS 6.0930 and 6.0933) and *E. faecalis* (KLDS 6.0934 and 6.0935) by 16S rRNA and *pheS* gene sequences respectively. The cholesterol removal mechanisms of all four *Enterococcus* strains in media were evaluated by measuring the cholesterol concentration of the supernatant fluid, washing fluid and fragmentized‐cells solution respectively. A part of cholesterol detected in the washing fluid was due to de‐conjugation of bile salts by bile salt hydrolase (BSH) activity of bacteria (Klaver and van der Meer, 1993; Grill *et al*., 2000; Franz *et al*., 2001). In general, BSH‐producing probiotic bacteria have a positive effect in lowering serum cholesterol levels through de‐conjugation of bile salts (Choi *et al*., 2014). Since the de‐conjugated bile salts are less absorbed in the intestine and excreted in the feces, the synthesis of new bile salts from cholesterol can reduce the total cholesterol concentration in the body. An amount of cholesterol retained in the cells was attributed to cholesterol assimilation of bacteria (Grill *et al*., 2000). The no‐recovered part of cholesterol was attributed to degradation of cholesterol by bacterial cells (Guo *et al*., 2011). The mechanisms of cholesterol removal from media were different in all assayed strains. For the strains of KLDS 6.0930, 6.0933 and 6.0935, the removal of cholesterol from media was explained by co‐precipitation of cholesterol with de‐conjugated bile salts, cell assimilation and cholesterol degradation, while cholesterol removal by the KLDS 6.0934 strain was due to both co‐precipitation and assimilation.

To be termed as a probiotic, a selected strain must reach alive to and colonize colon in abundant numbers. Before reaching the intestinal tract, probiotic bacteria must first survive transit through the stomach (Henriksson *et al*., 1999). The conditions such as pH 2.0, pH 2.5 and pH 3.0 for 2 h were selected to examine the acid tolerance of the bacteria. All of the assayed bacterial strains could survive for 2 h at pH 3.0. However, the viability of most bacteria at pH 2.0 was significantly decreased (*P* < 0.05) after 2 h, except for *E. durans* KLDS 6.0930, which was the most acid‐tolerant strain. In the small intestine, the most serious obstacle for probiotic survival is bile salts. 0.3% oxgall was used to examine the bacterial bile tolerance. All of the tested strains were grown better in MRS broth supplemented with oxgall. Moreover, the strain *E. durans* KLDS 6.0930 exhibited the highest tolerance to bile, because it could require a less time to increase by 0.3 units in absorbance at 620 nm than other strains exposed to oxgall. It is generally believed that the organism to live permanently in the host's intestine must be able to attach to intestinal mucosal cells (O'Sullivan *et al*., 1992). Caco‐2 cells are human intestinal cell lines expressing morphologic and physiologic characteristics of normal human enterocytes (Brassart *et al*., 1998) that have been exploited to select and assess probiotics on the basis of their adhesion properties (Deepika *et al*., 2012; Kaewnopparat *et al*., 2013; Rao *et al*., 2013; Wang *et al*., 2013). The adhesion ability of *E. faecalis* KLDS 6.0934 to Caco‐2 cells were significantly stronger than that of other tested strains, but this strain was sensitive to acid. Therefore, the strain KLDS 6.0930 was selected for the animal feeding trials, mainly due to its high cholesterol removal ability in media and acid and bile resistant.

It is necessary to evaluate the safety of natural isolates from traditional homemade products, although enterococci have important implications in dairy industry, particularly in variety products of cheese (Giraffa, 2003). The most important factor for the safety evaluation of *Enterococcus* strains is their resistance against different antibiotics, particularly glycopeptides such as vancomycin (Klein, 2003). Various opinions exist as to whether it might be desirable that some probiotic strains show resistance to specific antibiotics. On the other hand, some lactic acid bacteria have the potential to serve as a host for antibiotic resistance genes and to transfer these genes to intestinal pathogenic bacteria (Curragh and Collins, 1992; Werner *et al*., 2013). However, according to previous studies (Salminen *et al*., 1998; Danielsen and Wind, 2003; Hollenbeck and Rice, 2012), many of these antibiotic resistance observed were considered to be intrinsic or natural resistance and non‐transmissible. Probiotic bacteria that have intrinsically antibiotic‐resistant traits may be an advantage in the clinical application. They can be given at the same time as the antibiotic treatment (Cebeci and Gürakan, 2003). All the strains assayed in this experiment were found to be sensitive to clinically important antibiotics (ampicillin, erythromycin and penicillin G). In general, the resistance to some antibiotics might be species specific. Although *E. faecalis* KLDS 6.0934 and KLDS 6.0935 showed resistance to vancomycin (MICs ≥ 1024 μg ml^‐1^), the respective resistance genes (*vanA* and *vanB*) were not found in these strains. Similarly, *E. durans* KLDS 6.0930 and KLDS 6.0933 were phenotypically resistant to chloramphenicol (MIC of 16 μg ml^‐1^), but the two strains did not contain the gene *cat*_pIP501_. Moreover, all tested *Enterococcus* strains exhibited MICs ≥ 1024 μg ml^‐1^ to gentamycin, but no resistance gene [*aac(6′)‐aph(2′)‐Ia*] was observed. In this respect, the phenotypic resistance of *Enterococcus* strains to vancomycin or gentamycin might be attributed to other genes which were not detected in this paper (Hollenbeck and Rice, 2012), such as genes (*vanH*, *vanY*, *vanX*, *vanS* and *vanR*) responsible for vancomycin resistance and several other genes [*aph(2″)‐Ic*, *aph(2″)‐Id* and *aph(2″)‐Ib*‘] responsible for gentamycin resistance.

High level of serum cholesterol is a major risk factor for CHD in humans. Administration of lactic acid bacteria, including certain strains of the genus *Enterococcus*, has been claimed to decrease the concentration of serum cholesterol in humans and/or animals (Kiessling *et al*., 2002; Hlivak *et al*., 2005; Cavallini *et al*., 2009; Jones *et al*., 2012; Kumar *et al*., 2013). In the present study, *E. durans* KLDS 6.0930 was selected and administrated in rats fed on a high‐cholesterol diet. The results revealed that a decrease in serum TC and LDL‐C levels were observed (*P* < 0.05) after 4 weeks of *E. durans* KLDS 6.0930 administration, but there was no significant impact on serum TG and HDL‐C levels (*P* > 0.05) compared with the high‐cholesterol diet control group. These findings were consistent with previously published papers (Agerholm‐Larsen *et al*., 2000; Hlivak *et al*., 2005) that individuals or animals had a reduction in serum TC or LDL‐C levels when supplied with *Enterococcus* strains or their fermented products. In this study, more total bile acid in the feces was observed in *E. durans* KLDS 6.0930‐treated rats compared with the control group fed a diet rich in cholesterol. Bile salt hydrolase‐producing probiotic lactic acid bacteria might influence serum cholesterol by the promotion of fecal bile acid excretion (Pavlović *et al*., 2012). For this reason, the reduction of serum cholesterol levels in rats probably be attributed to the BSH activity of *E. durans* KLDS 6.0930, according to the findings in Table 1.

In conclusion, the results of this study showed that two strains of *E. durans* (KLDS 6.0930 and 6.0933) and two strains of *E. faecalis* (KLDS 6.0934 and 6.0935) were found to possess desirable probiotic properties *in vitro*. With regard to the strain *E. durans* KLDS 6.0930 showed cholesterol‐lowering effects in rats fed a cholesterol‐enriched diet for 4 weeks. This strain can be considered as a good candidate of potential cholesterol‐lowering agent.

## Materials and methods

### Isolation and selection of cholesterol‐lowering *E* *nterococcus* strains

*Enterococcus* strains were isolated according to Guo and colleagues (2011), and the isolates showed catalase negative and Gram‐positive were selected for further studies. *Enterococcus* isolates were grown in the MRS broth supplemented with cholesterol at a final concentration of 100 μg ml^‐1^ to evaluate their cholesterol removal abilities (Guo *et al*., 2011). The concentrations of cholesterol in supernatant fluid, washing fluid and the solution of fragmentized cells were determined respectively. And the cholesterol removal (CR) and cholesterol degradation ratio (CDR) were calculated and assessed according to Guo and colleagues (2011).

### Identification of the isolates by 16S rRNA and *pheS* gene sequences

The isolates were identified by the analysis of 16S rRNA and *pheS* gene sequences. The nucleic acid of the bacteria was extracted according to the manufacturer's instruction of the DNA extraction kit (Tiangen, Beijing, China). The 16S rRNA gene was amplified by the universal primers, 27F and 1541R (Artursson and Jansson, 2003). The amplification of *pheS* gene was performed with primers (pheS‐21‐F and pheS‐22‐R) according to Naser and colleagues (2005). The amplified productions were sequenced (BGI‐Shengen, China), and then the phylogenetic trees were created based on the 16S rRNA and *pheS* gene sequences using the neighbour‐joining methods by the mega 5.1 software (Tamura *et al*., 2011). The reliability of the groups was evaluated by bootstrap with 1000 re‐samplings. The gene sequences of 16S rRNA and *pheS* for type strains were obtained from the National Center for Biotechnology Information database. And the gene sequences of isolates were also submitted to GenBank under accession numbers KF768355, KJ818114, KJ818113 and KJ818115 (16S rRNA gene), and KF776544, KJ818116, KJ818117 and KJ818118 (*pheS* gene).

### Acid tolerance

The isolates grown in MRS broth were subcultured at least three times before the assay. Bacterial cells from overnight (18 h) cultures were harvested (12000 × g, 10 min, 4^o^C) and washed twice with sterile saline buffer (0.85%), and then were re‐suspended in MRS broth adjusted by HCl to pH 2.0, pH 2.5 and pH 3.0, respectively, and incubated anaerobically at 37^o^C for 2 h. Viability was determined using the plate count method. One millilitre sample was taken under the sterile condition at 0 h, 1 h and 2 h, and 10‐fold serial dilutions were made using sterile saline buffer (0.85%). The dilutions were plated on MRS agar and incubated anaerobically at 37^o^C for 24 h before enumeration.

### Bile tolerance

Each strain was subcultured at least three times before the assay, and then the isolates were incubated in MRS broth added 0.3% (w/v) oxgall powder (Amresco, USA) at 37^o^C under anaerobic condition. Bacterial growth was monitored by measuring absorbance with a spectrophotometer (DU 800, Beckman Coulter, USA) at 620 nm at hourly intervals for 8 h. The absorbance values obtained were plotted against the incubation time, and the bile tolerance of each strain was based on the time required for the absorbance value to increase by 0.3 units. The isolates incubated in MRS broth without oxgall powder were taken as the control.

### Adherence to Caco‐2 cells *in vitro*

The epithelial intestinal cell line Caco‐2 was employed for the adhesion assay of the isolates (Jacobsen *et al*., 1999). They were cultured in high‐glucose Dulbecco's Modified Eagle's Medium (DMEM; Gibco, USA) supplemented with 10% (v v^‐1^) heat‐inactivated (56 ^o^C for 30 min) fetal bovine serum, 100 U ml^‐1^ penicillin and 100 mg ml^‐1^ streptomycin. Cells were seeded in 6‐well tissue culture plates at a concentration of 10^5^ cells well^‐1^ (COSTAR, USA). The cell culture medium in the wells was replaced with fresh medium every other day. Cells were used for adhesion assays at late post‐confluence. The Caco‐2 cells were washed twice with PBS (pH 7.2) before bacterial cells were added (Le Blay *et al*., 2004). Approximately, 10^9^ cfu ml^‐1^ of each strain were re‐suspended in 2 ml DMEM without antibiotics, and added to the different wells. The plates were incubated at 37 ^o^C for 2 h in the condition of 5% CO_2_ and 95% air, and then the monolayer was washed three times with PBS. One millilitre of 0.25% trypsin‐EDTA solution (Gibco, USA) was added to each well, and then incubated 15 min at 37^o^C. One millilitre of DMEM supplemented with 10% bovine serum was added to each well to terminate the activity of trypsin. The serial 10‐fold dilutions of cell suspensions were plated on MRS agar to determine the number of adherent bacterial cells. The MRS plates were incubated at 37 ^o^C for 24 h. The results of the adhesion assay were expressed as a percentage, i.e. the ratio between the number of bacterial cells remained and the total number of bacterial cells added initially to each well.

### Antibiotic susceptibility test

For testing antibiotic resistance, bacterial strains were inoculated (1%, v v^‐1^) in MRS broth supplemented with different antibiotics at various final concentrations (1, 2, 4, 8, 16, 32, 64, 128, 256, 512 and 1024 μg ml^‐1^), including ampicillin, vancomycin, gentamicin, erythromycin, chloramphenicol and penicillin (Amresco, USA). The MICs of a given antibiotic was visually evaluated as the lowest concentration at which no growth was observed following a 24 h incubation period at 37^o^C. Interpretation for susceptibility status was based on the criteria adopted by European Food Safety Authority in the assessment of bacterial resistance to antibiotics (European Food Safety Authority, 2008). The possible presence of genes responsible for resistance to gentamicin [*aac(6′)‐aph(2′)‐Ia*], chloramphenicol (*cat*_pIP501_) and vancomycin (*vanA* and *vanB*) was investigated for the four isolates by PCR assays. Primers and protocols were described by Maietti and colleagues (2007).

### Animals and diets

Twenty‐four male Sprague‐Dawley (SD) rats aged 6 weeks were obtained from the Animal Research Centre of Jilin University, Changchun, China. The rats were fed a regular chow diet. The high‐cholesterol diets were consisted of 1% cholesterol, 10% lard, 5% sucrose, 0.3% sodium cholate and 83.7% regular chow. All rats were housed in a controlled animal room at 20 ^o^C and a relative humidity of 50% and maintained on a 12 h light‐dark cycle. Feed and water were provided daily ad libitum. After a 1‐week adaptation to regular chow diet, rats were randomly divided into three groups (each group *n* = 8): (i) a normal diet (ND) control group, regular chow diet + sterilized saline, (ii) a high‐cholesterol diet (HD) control group, high‐cholesterol diet + sterilized saline and (iii) a HD with KLDS 6.0930 group, high‐cholesterol diet + *E. durans* KLDS 6.0930. The strain *E. durans* KLDS 6.0930 was suspended in sterilized saline to a level of 2 × 10^9^ cfu ml^‐1^ before administration. During the 4‐week study period, three groups intra‐gastrically received sterilized saline or *E. durans* KLDS 6.0930 suspensions at a dosage of 1 ml 100g^‐1^ body weight in each day. Food intake and body weight were recorded daily throughout the feeding trial.

### Analysis of serum lipids

After 28 days of gavage, the rats were fasted for 12 h. Blood samples were collected and centrifuged immediately at 1500 × g for 15 min at 4 ^o^C to obtain serum. Serum was analysed for TC, TG, LDL‐C and HDL‐C using commercial kits (Kehua Bio‐engineering, Shanghai, China).

### Determination of fecal bile acid

The faecal droppings of the experimental rats were collected over a 2‐day period (days 27 and 28). Then, the faecal samples were dried and ground to a fine powder for further analysis. Fecal bile acid was determined using a bile acid assay kit (Kehua Bio‐engineering, Shanghai, China).

### Statistical analysis

The results are expressed as mean ± standard deviation. Statistical analyses of the data were performed using spss 17.0 software (SPSS, Chicago, IL, USA). One‐way analysis of variance was employed to evaluate the experimental data. A *P* value < 0.05 was considered statistically significant.

## Conflict of interest

None declared.

## Supporting information


**Fig. S1.** Neighbour‐joining tree showing the phylogenetic relationships of strain KLDS 6.0930, strain KLDS 6.0933, strain KLDS 6.0934, strain KLDS 6.0935 and related‐type strains based on the 16S rRNA gene sequences. *Listeria monocytogenes* was included as an outgroup.
**Fig. S2.** Neighbour‐joining tree showing the phylogenetic relationships of strain KLDS 6.0930, strain KLDS 6.0933, strain KLDS 6.0934, strain KLDS 6.0935 and related‐type strains based on the *pheS* gene sequences. *Ralstonia solanacearum* was included as an outgroup.
