# Supplementary material for: Probiotic properties of E nterococcus strains isolated from traditional naturally fermented cream in China
Source: Microb Biotechnol. 2015 Jul 22;9(6):737–45. doi: 10.1111/1751-7915.12306 (PMC5072190; doi:10.1111/1751-7915.12306)
Supplement: Supplementary file 1 — Fig. S1. Neighbour‐joining tree showing the phylogenetic relationships of strain KLDS 6.0930, strain KLDS 6.0933, strain KLDS 6.0934, strain KLDS 6.0935 and related‐type strains based on the 16S rRNA gene sequences. Listeria monocytogenes was included as an outgroup. Fig. S2. Neighbour‐joining tree showing the phylogenetic relationships of strain KLDS 6.0930, strain KLDS 6.0933, strain KLDS 6.0934, strain KLDS 6.0935 and related‐type strains based on the pheS gene sequences. Ralstonia solanacearum was included as an outgroup. [file MBT2-9-737-s001.doc]

*Enterococcus durans* DSM20633^T^ (AJ276354)

KLDS 6.0933 (KJ818114)

KLDS 6.0930 (KF768355)

*Enterococcus hirae* DSM 20160^T^ (Y17302)

*Enterococcus faecium* LMG 11423^T^ (AJ301830)

*Enterococcus lactis* BT159^T^ (GU983697)

*Enterococcus villorum* LMG 12287^T^ (AJ271329)

*Enterococcus viikkiensis* IE3.2^T^ (HQ378515)

*Enterococcus avium* ATCC 14025^T^ (AF133535)

*Enterococcus raffinosus* NCIMB 12901^T^ (Y18296)

*Enterococcus asini* AS2^T^ (Y11621)

*Enterococcus dispar* ATCC51266^T^ (AF061007)

*Enterococcus aquamarinus* LMG 16607^T^ (AJ877015)

*Enterococcus saccharolyticus* ATCC 43076^T^ (AF061004)

*Enterococcus sulfureus* ATCC49903^T^ (AF061001)

*Enterococcus caccae* 2215-02^T^ (AY943820)

*Enterococcus ureasiticus* CCRI-16986^T^ (GU457264)

*Enterococcus ureilyticus* CCM4629^T^ (AJ276352)

*Enterococcus plantarum* CCM 7889^T^ (HQ847537)

*Enterococcus rivorum* HAMBI 3055^T^ (FN822765)

KLDS 6.0934 (KJ818113)

*Enterococcus faecalis* JCM5803^T^ (AB012212)

KLDS 6.0935 (KJ818115)

*Listeria monocytogenes* NCTC10357^T^ (X56153)

91

100

83

79

100

90

99

57

81

86

78

76

61

98

98

88

86

83

58

94

0.01

**Fig. S1** Neighbour-joining tree showing the phylogenetic relationships of strain KLDS 6.0930, strain KLDS 6.0933, strain KLDS 6.0934, strain KLDS 6.0935 and related type strains based on the 16S rRNA gene sequences. *Listeria monocytogenes* was included as an outgroup.

KLDS 6.0934 (KJ818117)

KLDS 6.0935 (KJ818118)

*Enterococcus faecalis* LMG 7937^T^ (AJ843387)

*Enterococcus phoeniculicola* DSM 14726^T^ (AJ843394)

*Enterococcus rivorum* HAMBI 3055^T^ (FN870421)

*Enterococcus villorum* LMG 12287^T^ (AJ843378)

*Enterococcus ratti* LMG 21828^T^ (AJ843386)

*Enterococcus hirae* LMG 6399^T^ (AJ843420)

*Enterococcus haemoperoxidus* LMG 19487^T^ (AJ843415)

*Enterococcus caccae* CCM 7399^T^ (FR731111)

*Enterococcus moraviensis* LMG 19486^T^ (AJ843422)

*Enterococcus mundtii* LMG 10748^T^ (AJ843374)

*Enterococcus faecium* LMG 11423^T^ (AJ843428)

*Enterococcus durans* LMG 10746^T^ (AJ843373)

KLDS 6.0930 (KF776544)

KLDS 6.0933 (KJ818116)

*Enterococcus gallinarum* LMG 13129^T^ (AJ843464)

*Enterococcus sulfureus* LMG 13084^T^ (AJ843427)

*Enterococcus aquimarinus* LMG 16607^T^ (AJ969418)

*Enterococcus dispar* LMG 13521^T^ (AJ843382)

*Enterococcus avium* LMG 10744^T^ (AJ843457)

*Enterococcus raffinosus* LMG 12888^T^ (AJ843381)

*Enterococcus asini* LMG 18727^T^ (AJ843430)

*Ralstonia solanacearum* CMR15 (FP885895)

100

100

57

66

81

78

61

29

24

40

26

26

9

10

7

4

9

3

2

0.1

**Fig. S2** Neighbour-joining tree showing the phylogenetic relationships of strain KLDS 6.0930, strain KLDS 6.0933, strain KLDS 6.0934, strain KLDS 6.0935 and related type strains based on the *pheS* gene sequences. *Ralstonia solanacearum* was included as an outgroup.
